# Physiological and transcriptomic responses of *Ginkgo biloba* to salt stress and exogenous melatonin

**DOI:** 10.3389/fpls.2026.1881953

**Published:** 2026-08-03

**Authors:** Xinyu Yang, Shurong Peng, Yanxian Zhao, Dongmei Shi, Fusheng Wu, Limin Sun, Qian Zhang

**Affiliations:** 1 Key Laboratory of National Forestry and Grassland Administration for Warm Temperate Forest Ecosystem Conservation and Restoration, Forestry College of Shandong Agricultural University, Tai’an, Shandong, China; 2 Tai’an 88th Hospital, Tai’an, Shandong, China; 3 Weifang Engineering Vocational College, Weifang, Shandong, China; 4 Shandong Provincial Center for Forest and Grassland Germplasm Resources, Jinan, Shandong, China; 5 Shandong Academy of Forestry Sciences, Jinan, Shandong, China

**Keywords:** exogenous melatonin, flavonoids, *Ginkgo biloba*, MAPK, photosynthesis, salt stress

## Abstract

**Introduction:**

Soil salinization constrains plant growth, and enhancing salt tolerance is of great significance.

**Methods:**

Here, *Ginkgo biloba* seedlings were treated with control (CK), salt stress (S), or salt stress + melatonin (S+M). Integrating physiology, transcriptomics, and weighted gene co-expression network analysis (WGCNA), we explored how melatonin enhances salt tolerance.

**Results and Discussion:**

Salt stress reduced chlorophyll content, suggesting potential impairment of photosynthetic capacity, appeared to increase membrane lipid peroxidation, and led to substantial proline accumulation. Melatonin treatment appeared to up-regulate the MAPK gene *MYC2* and down-regulate *PP2C* and *RbohD*, suggesting a potential alleviation of oxidative stress. WGCNA identified MEblue (MAPK-enriched) positively correlated with salt stress and proline, and MEgreen (photosynthesis-enriched) positively correlated with chlorophyll and catalase (CAT) activity but negatively correlated with salt stress. Melatonin appeared to help restore MEgreen expression, potentially reducing oxidative damage and appearing to help stabilize chlorophyll. Melatonin also appeared to help maintain photosystem II (PSII) genes (*Lhcb1*, *Lhcb4*) and reduced flavonoid accumulation. These findings provide insights into the model of melatonin-mediated salt stress responses in gymnosperms. This work may support salt-tolerant *Ginkgo biloba* breeding and the application of melatonin in forestry.

## 1 Introduction

Among the various abiotic stresses, salt stress stands out as a major factor restricting global plant growth and crop productivity (Lu et al., 2025). It exerts multifaceted detrimental effects on plants, encompassing physiological and metabolic dysregulation, impairment of cellular function, and disruption of hormonal homeostasis, thereby posing a substantial threat to the sustainability of agricultural production and the stability of ecological systems (Liu et al., 2024; Tibesigwa et al., 2025). Under saline conditions, elevated salt concentrations induce osmotic stress by reducing cellular water potential and inhibiting water uptake, which in turn triggers stomatal closure (Zhao et al., 2020). Moreover, salt stress disrupts cellular ion homeostasis, leading to the excessive accumulation of Na^+^ and Cl^-^ (García-Caparrós et al., 2018) and consequently interfering with critical metabolic processes such as photosynthesis and enzyme activation (Ma et al., 2026). High salt stress disrupts electron transport in organelles, causing ROS burst and subsequent oxidation of lipids and proteins (Zhou et al., 2024). Furthermore, salt stress perturbs phytohormone balance, elevating abscisic acid (ABA) levels to modulate stomatal responses while suppressing cytokinin activity. These hormonal alterations collectively contribute to growth inhibition, foliar chlorosis, and abscission (Singh and Roychoudhury, 2023; Shafi et al., 2025).

Melatonin (N-acetyl-5-methoxytryptamine) is an important endogenous molecule with diverse functions in plants (Reiter et al., 2015; Xu et al., 2024). In plants, melatonin functions as a key modulator within stress-responsive signaling pathways, acting to directly scavenge free radicals, promote the activation of antioxidant enzymes, and preserve the functional integrity of the photosynthetic apparatus (Sun et al., 2021; Singh et al., 2023). Melatonin also improves plant abiotic stress tolerance via modulation of secondary metabolites (e.g., flavonoids, isoflavonoids) and hormone crosstalk (Yin et al., 2022; Jindal et al., 2024). In salt-exposed Silybum marianum L., foliar-applied melatonin relieved ROS-triggered oxidative damage by modulating stomatal aperture, internal CO_2_ levels, and photosynthetic capacity (Kang et al., 2024). In pomegranate, melatonin alleviates membrane lipid peroxidation and preserves membrane permeability through the activation of antioxidant enzymes (superoxide dismutase, SOD; peroxidase, POD; and catalase, CAT) and the upregulation of genes associated with osmotic adjustment (Yang et al., 2025). Moreover, melatonin fortifies pear resistance to Botryosphaeria dothidea by activating the antioxidant system and the phenylpropanoid metabolic pathway, while concurrently modulating jasmonic acid and phloridzin levels (Xu et al., 2024).

A renowned “living fossil,” *Ginkgo biloba* represents the most ancient gymnosperm still in existence, offering diverse applications in medicine, nutrition, wood products, and ornamental horticulture (Crane, 2019; Liu et al., 2022). In recent decades, escalating soil salinization has emerged as a critical constraint on its cultivation and growth. However, certain intrinsic traits, such as low stomatal density and a likely low proportion of xylem conduits, may render its photosynthetic function comparatively less sensitive to soil salinity (Matsuura et al., 2026). Notably, exogenous application of melatonin has been demonstrated to effectively ameliorate salt-induced injury and enhance salt tolerance in this species (Zhou et al., 2024). However, the transcriptional regulatory mechanisms through which melatonin mitigates salt stress in G. *biloba* remain poorly elucidated. In the present study, we utilized G. *biloba* seedlings as the experimental model and applied melatonin via a combined foliar spray and root drench approach to monitor physiological dynamics under saline conditions. By integrating comprehensive transcriptome profiling with physiological assessments, we further delineate the potential mechanisms underlying melatonin-mediated alleviation of salt stress in G. *biloba*. This investigation aims to establish a foundational framework for advancing research on salt tolerance in woody perennials and to promote the practical deployment of melatonin in forestry applications. Furthermore, it seeks to provide critical insights into the enhancement of ecological adaptability in G. *biloba* and to furnish supplementary evidence that enriches our understanding of salt tolerance mechanisms in gymnosperms.

## 2 Materials and methods

### 2.1 Experimental materials

In this study, one-year-old half-sib family seedlings of *Ginkgo biloba* were used as the experimental material. Seeds were collected in September 2024 from the Daizong Campus of Shandong Agricultural University, and after stratification in moist sand to promote germination, they were sown in pots in late March 2025 (substrate: soil = 2:1). Seedlings were maintained under natural environmental conditions until August, when uniformly vigorous individuals were selected and randomly allocated to three treatment groups: control group (CK), salt stress group (S), and salt stress + melatonin group (S+M). For each treatment, 21 seedlings were planted in 7 pots (3 seedlings per pot). At each sampling time point, seedlings from one pot were collected, with each of the 3 seedlings serving as one biological replicate (n= 3 per time point per treatment). All plants were grown under natural outdoor conditions throughout the experiment, with an average daily temperature of approximately 27 °C. Sampling was performed at seven time points throughout the experimental period: 0, 5, 10, 15, 20, 25, and 30 days post-treatment (d).

### 2.2 Salt stress treatment

The experiment lasted 40 days in total. After a 10-day pre-treatment with 0.1% NaCl (Days 0–10), day 10 was set as the baseline and designated as Day 0 for the formal experimental period. All time points shown in the figures (Days 0, 5, 10, 15, 20, 25, and 30) are based on this baseline. The formal experiment was then divided into two phases: 0.1% NaCl treatment (formal Days 0–15, corresponding to overall Days 10–25) and 0.3% NaCl treatment (formal Days 16–30, corresponding to overall Days 26–40). Both NaCl and melatonin treatments were applied on each sampling day (every 5 days) during the formal experimental period.

The NaCl concentrations (0.1% and 0.3%) and the melatonin concentration (100 μM) were selected based on preliminary experiments; the melatonin concentration was also supported by previous studies demonstrating its effectiveness in alleviating salt stress in *Ginkgo biloba* seedlings (Zhou et al., 2024). NaCl was applied at 0.1% and 0.3% of soil dry weight, with 200 mL of the corresponding solution per pot per sampling day. Soil EC was monitored in real time, and additional NaCl solution was supplemented as needed to maintain stable concentrations. The control group received the same volume of purified water to ensure consistent moisture conditions.

0-10d: Seedlings in the S and S+M groups were irrigated with 0.1% NaCl without melatonin to allow acclimation to salt stress, while the control group received purified water. No melatonin was applied to either group during this phase.

11-25d (formal Days 0–15): The S group continued under 0.1% NaCl, while the S+M group received 100 μM melatonin via foliar spray and root drench, with salt stress maintained as before. For root application, melatonin was mixed with the NaCl solution and applied as a drench (200 mL per pot). For foliar application, the same melatonin solution was sprayed onto the leaves until runoff (i.e., until droplets began to form on the leaf surface), without a fixed volume. Salt stress was maintained as before.

26-40d (formal Days 16–30): NaCl was increased to 0.3% of soil dry weight, corresponding to an EC of approximately 5000–6000 μS cm^-^¹. Treatment regimens for all groups continued as in the preceding phase.

### 2.3 Determination of physiological parameters

Chlorophyll content was quantified according to the method described by Lichtenthaler and Wellburn (1983). Relative electrical conductivity (REC) was determined following the procedure established by Nijabat et al. (2020). The activities of superoxide dismutase (SOD) (Catalog Number: G0101F), peroxidase (POD) (G0107F), and catalase (CAT) (G0105F), as well as the contents of malondialdehyde (MDA) (G0109F), proline (PRO) (G0111F), and flavonoids (G0118F), were measured using commercially available assay kits (Suzhou Grace Biotechnology Co., Ltd., Suzhou, China) in accordance with the manufacturer’s instructions. Absorbance values were recorded using a V-5600 spectrophotometer, and the corresponding concentrations were subsequently calculated.

### 2.4 Transcriptome sequencing and bioinformatics analysis

Samples collected on day 30 from the CK, S, and S+M treatment groups (three biological replicates per group) were selected for transcriptome sequencing. All samples were collected from leaves. Total RNA was extracted using a combination of CTAB and PBIOZOL methods. RNA quality and concentration were assessed using a Qubit fluorometer and a Qsep400 fragment analyzer. mRNA was enriched using Oligo(dT) magnetic beads, fragmented, and reverse-transcribed into cDNA using random hexamer primers. Strand-specific libraries were generated by incorporating dUTP during second-strand synthesis. After end repair, dA-tailing, adapter ligation, and PCR amplification, the libraries were circularized and amplified into DNA nanoballs (DNBs), which were then loaded onto the sequencing chip. Sequencing was performed on the MGI DNBSEQ-T7 platform with 150 bp paired-end reads. Sample correlation heatmap and PCA were generated to evaluate data repeatability (**Supplementary Figures 1**, **2**). Transcriptomic data were analyzed according to the method described by Feng et al. (2025). The reference genome of *Ginkgo biloba* was obtained from the GigaDB database (http://gigadb.org/dataset/100209), from which all gene IDs were derived. KEGG pathway assignments were based on the annotation results provided by the sequencing company, and the pathway diagrams followed the KEGG nomenclature (**Supplementary Figure 5**, obtained from the KEGG database; reproduced with permission from Kanehisa et al., 2025).

### 2.5 Quantitative real-time PCR analysis

Total RNA was extracted from 0.3% NaCl-stressed *Ginkgo biloba* leaves harvested at three time points. The samples were ground under cryogenic conditions, and RNA isolation was carried out using VeZol reagent (Vazyme Biotech Co., Ltd.). mRNA was then reverse transcribed into cDNA with the HiScript III First-Strand cDNA Synthesis Kit (+gDNA wiper) (Vazyme Biotech Co., Ltd.). *Ginkgo biloba* GAPDH served as an internal control (Liu et al., 2022), and gene-specific primers were designed using Primer Premier 5.0 software (**Supplementary Table 1**). qPCR was conducted on a CFX96 Real-Time PCR Detection System (Bio-Rad, Hercules, CA, USA) with ChamQ Blue Universal SYBR qPCR Master Mix (Vazyme Biotech Co., Ltd.). Relative transcript levels were determined via the 2^-^^ΔΔCt^ method.

### 2.6 Co-expression network analysis

WGCNA analysis was performed using the R package WGCNA. Low-expression genes and stably expressed genes across all samples were filtered using the varFilter function in the genefilter package to improve the accuracy of network construction. The optimal soft-thresholding power was selected using the pickSoftThreshold function, with an R² cut-off of 0.85, to ensure that the network satisfied the scale-free topology criterion. A weighted correlation network was constructed by raising the Pearson correlation coefficients to the selected power, enhancing strong correlations while suppressing weak or negative ones, ensuring that the gene connections followed a scale-free distribution. Modules were identified by hierarchical clustering based on gene expression correlation, where genes with similar expression patterns were assigned to the same module. Hub genes within each module were identified based on connectivity (degree) values.

### 2.7 Statistical analysis

Two-way ANOVA was carried out using GraphPad Prism 10.1.2. For multiple comparisons, we applied Tukey’s test (*p* < 0.05) via DPS 7.05 software. Figures were prepared with Adobe Illustrator 2024 and the Metware Cloud platform (https://cloud.metware.cn). Differentially expressed genes (DEGs) were identified using the criteria of |log_2_FC| ≥ 1 and *p* < 0.05, with false discovery rate (FDR) adjusted *q* < 0.05.

## 3 Results

### 3.1 Effects of exogenous melatonin on the phenotype and physiological parameters of *Ginkgo biloba* under salt stress

**Figure 1:**
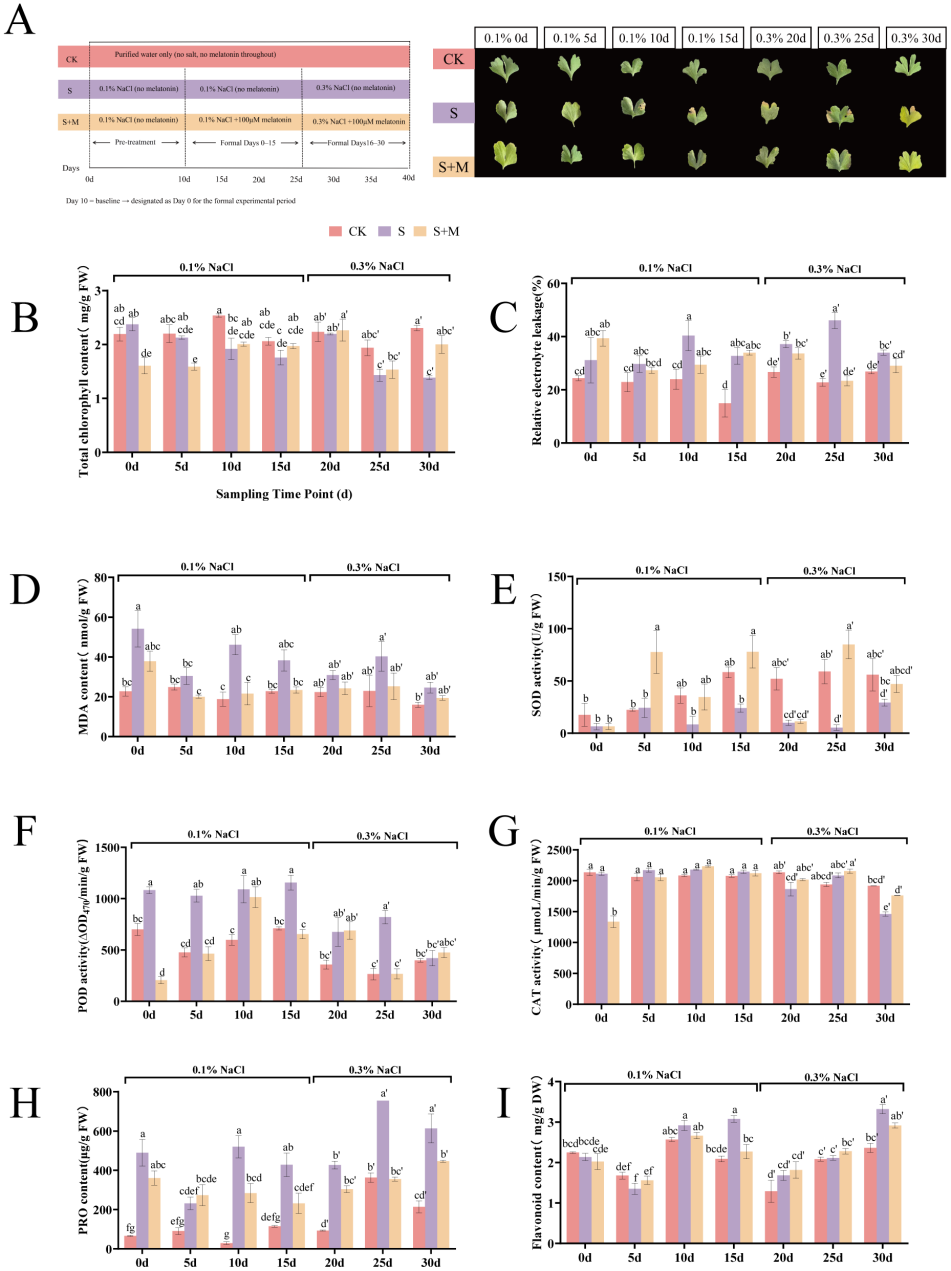
Effects of salt stress and melatonin treatment on the phenotype and physiological parameters of *Ginkgo biloba* leaves. **(A)** Schematic diagram of the experimental design (left) and leaf phenotype (right). CK, control (purified water only, no salt; red); S, salt stress (0.1% NaCl → 0.3% NaCl; purple); S+M, salt stress (0.1% NaCl → 0.3% NaCl) + 100 μM melatonin (yellow). Detailed experimental procedures are described in Section 1.2. Day 0 corresponds to the baseline after 10 days of pre-treatment with 0.1% NaCl; at this time, both S and S+M groups had received salt stress only, without melatonin application. **(B)** Total chlorophyll content; **(C)** Relative electrical conductivity (REC); **(D)** Malondialdehyde (MDA) content; **(E)** Superoxide dismutase (SOD) activity; **(F)** Peroxidase (POD) activity; **(G)** Catalase (CAT) activity; **(H)** Proline (PRO) content; **(I)** Flavonoid content. Days 0–15: 0.1% NaCl; days 20–30: 0.3% NaCl. Different lowercase letters (a, b) indicate significant differences under 0.1% NaCl at *p* < 0.05; primed letters (a′, b′) under 0.3% NaCl at *p* < 0.05. Tukey’s test. Data are mean ± SD (n = 3).

As illustrated by the phenotypic observations (**Figure 1A**), leaves of the CK group remained consistently green throughout the experimental period. In contrast, leaves of the S group exhibited progressive chlorosis concomitant with the duration of stress exposure and increasing salt concentration, culminating in near-complete yellowing under 0.3% salt concentration applied for 30 days. The extent of foliar yellowing in the S+M group appeared to be less severe than that observed in the S group.

Salt stress influenced the physiological indicators of *Ginkgo biloba* leaves, with the effects generally intensifying as external salt concentrations increased. Under 0.1% NaCl, total chlorophyll content showed a sustained decline (**Figure 1B**), which became more pronounced under 0.3% NaCl. Exogenous melatonin application appeared to mitigate chlorophyll degradation, helping maintain relatively higher chlorophyll levels throughout the stress period. Both relative electrical conductivity (REC) and MDA content consistently followed the pattern S > S+M > CK (**Figures 1C, D**). At day 25 of the 0.3% NaCl concentration treatment, melatonin treatment reduced MDA content by 37.4% relative to the S group, indicative of a substantial alleviation of membrane lipid peroxidation. Under 0.1% salt concentration, salt exposure provoked a sharp elevation in POD activity concomitant with SOD inactivation. In contrast, melatonin application enhanced CAT activity while stabilizing POD levels. At day 25 of the 0.3% salt concentration treatment, POD activity in the S group attained a peak that was 3.07- and 3.09-fold greater than that recorded in the S+M and CK groups, respectively. Concomitantly, melatonin treatment elevated SOD activity to 84.9 U g^-^ S+M group than in the S group throughout the experimental period (**Figures 1E–G**). Furthermore, throughout the entire treatment period, both proline and flavonoid contents consistently followed the order: S > S+M > CK (**Figures 1H, I**). Under 0.3% NaCl stress at day 25, proline accumulation in the S group surpassed that of the S+M and CK groups by 111.9% and 107.3%, respectively. At day 30, flavonoid content in the S group exceeded that of the S+M group by 13.9% and that of the CK group by 40.5%, whereas values in the S+M group remained consistently intermediate (**Figures 1H, I**).

### 3.2 Screening of differentially expressed genes and functional enrichment analysis

Transcriptome sequencing was performed on samples from the CK, S, and S+M treatment groups (three biological replicates per group), yielding a total of 101.2 Gb of clean data. Clean data per individual sample ranged from 9.59 to 13.38 Gb, corresponding to 63.94 million to 89.21 million valid reads. The Q30 score for all samples was ≥98%, and GC content remained stable between 44.3% and 44.8% (**Supplementary Table 2**). Differentially expressed genes (DEGs) were identified using the threshold criteria of *p* < 0.05 and |log_2_FC| ≥ 1. Pairwise comparisons among treatments (**Supplementary Figures 3A–D**) revealed 4,089 DEGs in the S vs. CK comparison (1,999 up-regulated and 2,090 down-regulated), 3,065 DEGs in the S+M vs. CK comparison (1,543 up-regulated and 1,522 down-regulated), and 2,184 DEGs in the S+M vs. S comparison (855 up-regulated and 1,329 down-regulated).

GO functional enrichment analysis (**Supplementary Figures 3E–G**) assigned annotations to a total of 5,598 DEGs across the three pairwise comparisons, with terms distributed among three principal categories: biological process, cellular component, and molecular function. The most enriched pathways in both the S vs. CK and S+M vs. CK comparisons were related to chitin, amino sugar, and glucosamine metabolism, whereas those in the S+M vs. S comparison were associated with ADP binding, NAD^+^ nucleosidase activity, and programmed cell death. Kyoto Encyclopedia of Genes and Genomes (KEGG) pathway enrichment analysis (**Supplementary Figures 3H–J**) revealed distinct pathway enrichment profiles among the three comparisons. Specifically, 13, 12, and 5 pathways were significantly enriched (*q* < 0.05) in the S vs. CK, S+M vs. CK, and S+M vs. S comparisons, respectively (**Supplementary Table 3**). In the S vs. CK group, significantly enriched pathways were predominantly associated with secondary metabolism (ko01110), metabolic pathways (ko01100), plant-pathogen interaction (ko04626), photosynthesis-antenna proteins (ko00196), and the MAPK signaling pathway (ko04016), indicating that salt stress substantially perturbed the photosynthetic apparatus, primary metabolism, and immune signaling. The S+M vs. CK comparison exhibited significant enrichment of secondary metabolic pathways such as flavonoid biosynthesis (ko00941). Notably, the S+M vs. S comparison highlighted enrichment primarily in plant-pathogen interaction (ko04626), the MAPK signaling pathway (ko04016), and secondary metabolism, suggesting that melatonin-mediated alleviation of salt stress may operate through the modulation of these pathways.

### 3.3 Genes associated with the plant MAPK signaling pathway

**Figure 2:**
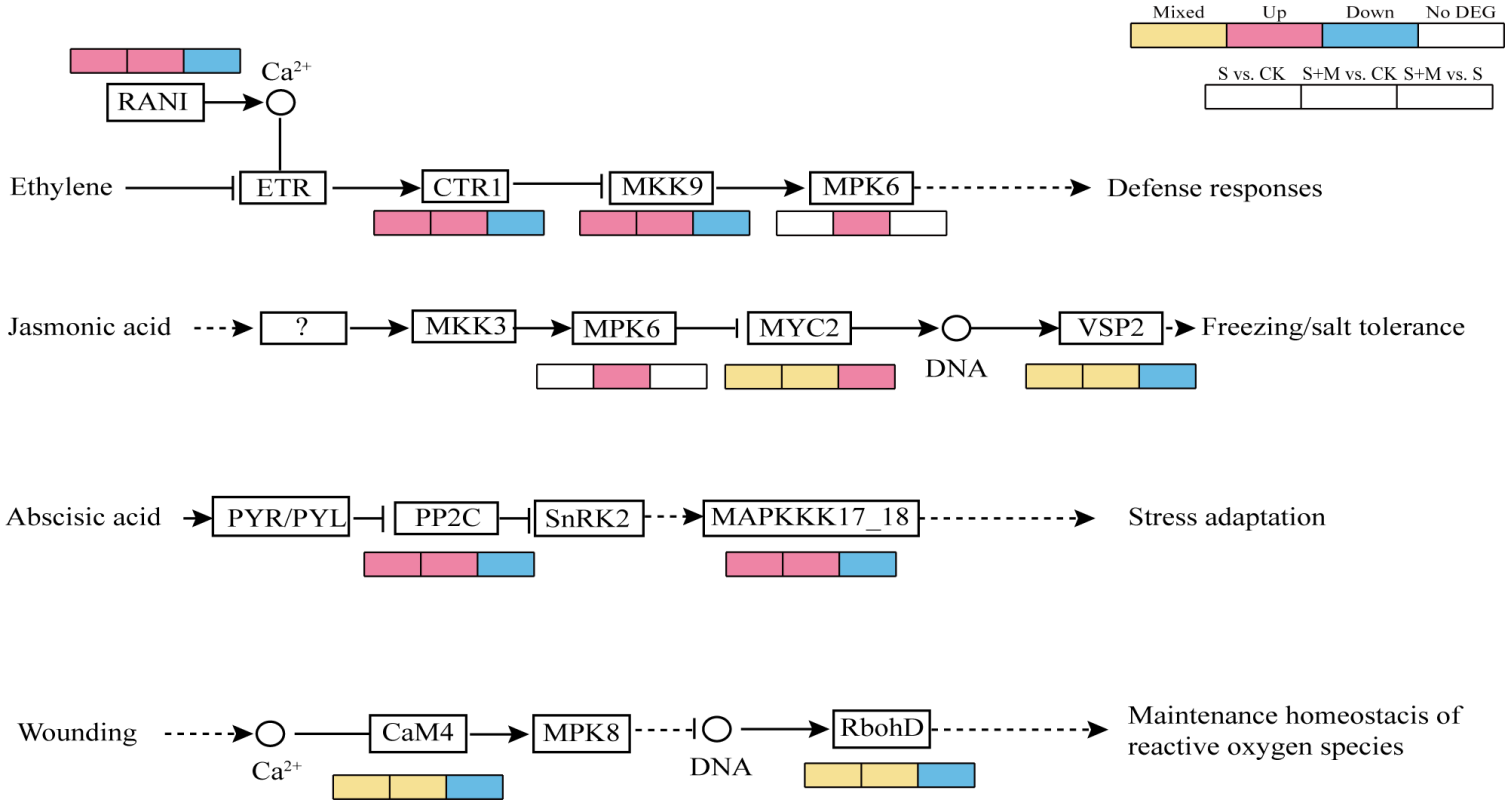
Simplified MAPK signaling pathway. Upstream signals include ethylene, JA, ABA, and wounding. Solid arrows: direct regulation; dashed arrows: indirect regulation or long-distance signal transduction; solid bars (——|): direct inhibition; dashed bars (-–|): indirect inhibition. Question marks ()?: upstream molecules not yet identified. The three white boxes show expression changes under S vs. CK, S+M vs. CK, and S+M vs. S. Yellow: both up- and down-regulation; pink: only up; blue: only down; blank: no DEGs. CK, S, and S+M are as in **Figure 1**. Detailed gene information is provided in **Supplementary Figure 4** and **Supplementary Table 4**.

By comparing gene expression profiles within the MAPK signaling pathway across the three pairwise comparisons, we delineated the regulatory mode by which melatonin modulates this pathway under salt stress (**Supplementary Figure 4**). A total of 133 DEGs were identified within this pathway (**Supplementary Table 4**), and a clustered heatmap is presented in **Supplementary Figure 1**. For simplicity, **Figure 2** presents the expression patterns of key regulatory nodes within the ethylene, jasmonic acid, abscisic acid, and wound-related signaling branches under three comparative contexts: salt stress alone (S vs. CK), salt stress + melatonin (S+M vs. CK), and the melatonin-mediated alleviation effect (S+M vs. S). Relative to the CK group, salt stress appeared to activate the MAPK cascade, as evidenced by significant up-regulation of upstream *MAPKKK17_18*, *MKK9*, and the negative regulator *PP2C*, whereas the expression patterns of the ROS-generating gene *RbohD* and the calcium sensor gene *CaM4* exhibited divergent trends. Upon melatonin application, most of these changes remained evident, with *MKK9, PP2C, MPK6*, and *MYC2* remaining up-regulated. In contrast, compared with salt stress alone, melatonin treatment significantly down-regulated the salt-induced expression of *MAPKKK17_18*, *MKK9*, and *PP2C*, concurrently suppressed the expression of *RbohD*, *CaM4*, and *VSP2*, and specifically up-regulated *MYC2* (*Gb_04728*; log_2_FC = 1.16), a core transcription factor in JA signaling.

### 3.4 Differential expression analysis of genes in the photosynthesis-antenna protein pathway

**Figure 3:**
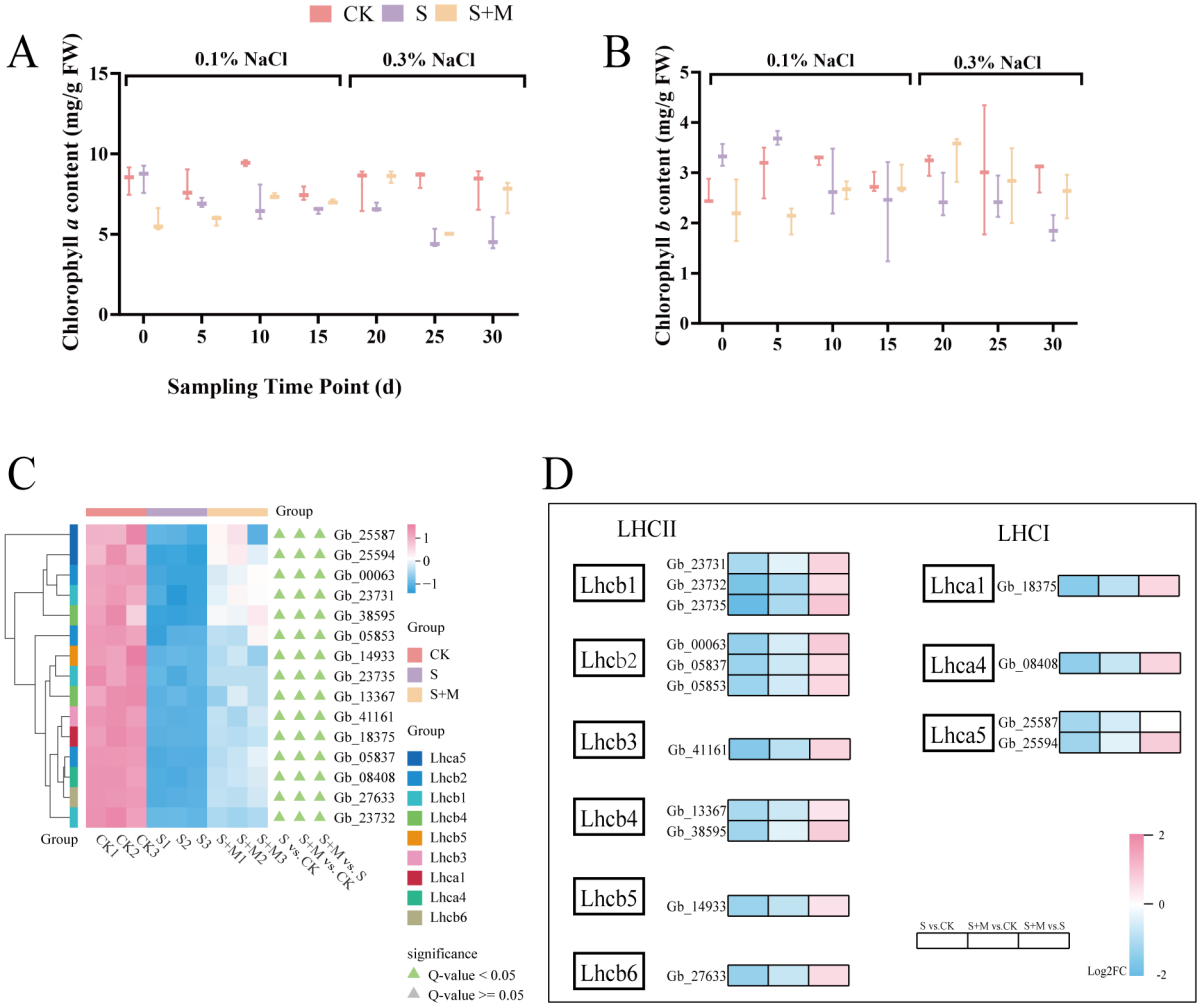
Chlorophyll content and photosynthesis-antenna protein pathway (ko00196) gene expression. **(A, B)** Chlorophyll a and b contents under 0.1% and 0.3% NaCl stress. CK, S, and S+M are as in **Figure 1**. Error bars: mean ± SD. **(C)** Z-score heatmap. Color codes are as in **Supplementary Figure 4**. **(D)** Pathway schematic with log_2_FC heatmaps for S vs. CK, S+M vs. CK, and S+M vs. S (pink: up; blue: down). Abbreviations are as in **Supplementary Figure 5**, which also provides a schematic diagram of the thylakoid membrane photosynthetic apparatus.

To investigate the protective mechanisms of melatonin on the photosynthetic system of *Ginkgo biloba* under salt stress, we first examined the dynamic changes in chlorophyll a (Chl a) and chlorophyll b (Chl b) contents. As shown in **Figure 3A, B**, salt stress treatment significantly reduced both Chl a and Chl b levels relative to the CK group, with the decline being more pronounced under high salt stress (0.3% NaCl). Notably, exogenous application of melatonin, particularly under the 0.3% NaCl regime, resulted in significantly higher Chl a and Chl b contents across all sampling time points compared with the S group, with values approaching those of the CK group. These results suggest that melatonin mitigates salt-induced inhibition of chlorophyll synthesis, implying a protective effect on the photosynthetic apparatus.

On this basis, we further analyzed the expression patterns of light-harvesting complex (LHC)-associated genes (**Supplementary Table 5**). The hierarchical clustering heatmap results (**Figure 3C**) revealed that the majority of LHC family genes exhibited high expression levels in the CK group. Salt stress treatment induced a pronounced global down-regulation of these genes, whereas melatonin application substantially restored their expression, resulting in an overall expression rank order of CK > S+M > S. Examination of individual gene expression patterns (**Figure 3D**) confirmed that, relative to the CK group, salt stress significantly suppressed key members of both the Lhca and Lhcb subfamilies, including *Lhca1*, *Lhca4*, *Lhca5*, and *Lhcb1–6*. Notably, melatonin treatment significantly rescued the expression of these genes; although the magnitude of up-regulation was moderate, the differences were statistically significant (*q* < 0.05). Collectively, these findings indicate that melatonin effectively alleviates the salt-induced transcriptional repression of photosynthesis-associated genes.

### 3.5 Differential expression analysis of genes in the flavonoid biosynthesis pathway

**Figure 4:**
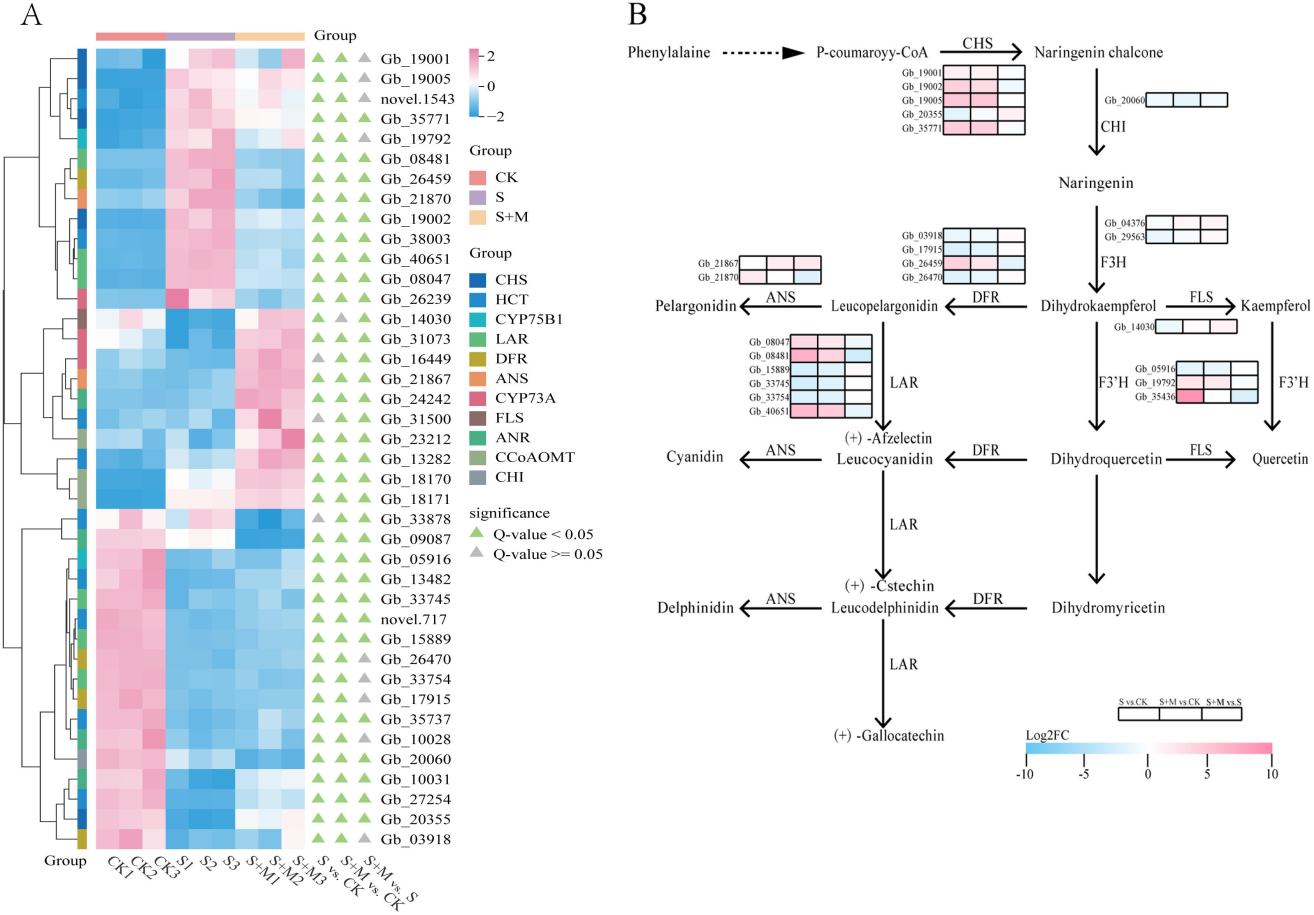
**(A)** Heatmap of flavonoid pathway DEGs (CK, S, S+M). Color codes are as in **Supplementary Figure 4**. Abbreviations (top to bottom): CHS, chalcone synthase; HCT, hydroxycinnamoyl transferase; CYP75B1, cytochrome P450 75B1 (flavonoid 3′-monooxygenase); LAR, leucoanthocyanidin reductase; DFR, dihydroflavonol 4-reductase; ANS, anthocyanidin synthase; CYP73A, cytochrome P450 73A (trans-cinnamate 4-monooxygenase/cinnamic acid 4-hydroxylase); FLS, flavonol synthase; ANR, anthocyanidin reductase; CCoAOMT, caffeoyl coenzyme A O-methyltransferase; CHI, chalcone isomerase. CK, S, and S+M are as in **Figure 1**. **(B)** Pathway schematic of flavonoid biosynthesis. Log_2_FC heatmaps for S vs. CK, S+M vs. CK, and S+M vs. **(S)** Color codes are as in **Figure 3D**. Abbreviations are as in **Figure 4A**. CK, S, and S+M are as in **Figure 1**.

Cluster analysis was performed on DEGs within the flavonoid biosynthesis pathway (ko00941) (**Figure 4A**). A total of 40 DEGs were identified within this pathway (**Supplementary Table 6**). In the CK group, genes associated with flavonoid biosynthesis exhibited relatively stable expression. Upon exposure to salt stress, genes of the anthocyanidin synthase (ANS) family were markedly up-regulated (*Gb_21867*; log_2_FC=1.83), whereas upstream *F3H*, *FLS* of the flavonol branch, and *ANR* of the proanthocyanidin branch were concurrently repressed. Following melatonin application, ANS family genes remained up-regulated, albeit with a diminished fold-change (*Gb_21867*; log_2_FC=1.73), while *DFR* and its downstream gene *F3’H* were down-regulated. Concomitantly, melatonin treatment significantly restored the expression of *CHS*, *F3H*, and *FLS*, all of which were suppressed by salt stress (**Figure 4B**), ultimately contributing to a reduction in total flavonoid content. Notably, among the ANR family members, *Gb_09087* exhibited the most pronounced down-regulation in the S+M vs. S comparison (log_2_FC = -4.60), suggesting that this gene may play a critical role in melatonin-mediated regulation of flavonoid metabolism.

### 3.6 Transcription factors

A total of 256 differentially expressed transcription factor-encoding genes (DETFs), distributed across 50 distinct families, were identified in this study. The five most abundant families comprised AP2/ERF-ERF (30 members), NAC (25 members), MYB (19 members), WRKY (19 members), and bHLH (15 members) (**Supplementary Figure 6A**). Pairwise comparisons among the three treatment groups revealed markedly distinct regulatory patterns of these transcription factors in response to the different experimental conditions.

Within the AP2/ERF-ERF family (**Supplementary Figure 6B**), the S vs. CK, S+M vs. CK, and S+M vs. S comparisons identified 11, 10, and 1 up-regulated genes, and 11, 6, and 10 down-regulated genes, respectively. Notably, *ERF1A* (*Gb_20395*) exhibited the highest expression levels in both the S and S+M treatment groups. *ERF5* (*Gb_34849*) was significantly up-regulated in the S+M vs. S comparison (log_2_FC = 3.36) but exhibited a down-regulated pattern in the S vs. CK comparison (log_2_FC=-2.76).

The NAC family exhibited a predominant trend of up-regulation across the three pairwise comparisons (**Supplementary Figure 6C**), with 22, 12, and 1 up-regulated genes and 2, 0, and 10 down-regulated genes identified in the S vs. CK, S+M vs. CK, and S+M vs. S comparisons, respectively. *NAC56* (*Gb_02849*) was consistently expressed at high levels across all three treatment groups and was significantly up-regulated in the S+M vs. CK comparison (log_2_FC = 1.66). Notably, in the S+M vs. S comparison, only *NAC79* (*Gb_01375*; log_2_FC=1.37) exhibited significant up-regulation.

The MYB family was predominantly down-regulated in the S vs. CK and S+M vs. CK comparisons. Specifically (**Supplementary Figure 6D**), 0 up-regulated and 11 down-regulated genes were detected in the S vs. CK comparison, whereas 3 up-regulated and 4 down-regulated genes were observed in the S+M vs. CK comparison. In the S+M vs. S comparison, however, 4 genes were up-regulated and 3 were down-regulated. For instance, *MYB27* (*Gb_23187*), *MYB39* (*Gb_09907*), and *MYB14* (*Gb_33428*) were all up-regulated in the S+M vs. S comparison but exhibited down-regulation in the S vs. CK comparison.

The WRKY family was predominantly up-regulated in both the S vs. CK and S+M vs. CK comparisons, with 9 up-regulated and 2 down-regulated genes detected in the former, and 13 up-regulated and 7 down-regulated genes in the latter (**Supplementary Figure 6E**). In the S+M vs. S comparison, only *WRKY46* (*Gb_36273*; log_2_FC=3.36) exhibited significant up-regulation.

The bHLH family displayed a markedly treatment-specific expression pattern (**Supplementary Figure 6F**). In the S vs. CK comparison, all seven differentially expressed genes were down-regulated. The S+M vs. CK comparison yielded an equal distribution of five up-regulated and five down-regulated genes. In the S+M vs. S comparison, only two differentially expressed genes were identified: *bHLH062*, which was up-regulated, and *bHLH067*, which was down-regulated.

### 3.7 Co-expression module identification and core gene network construction

**Figure 5:**
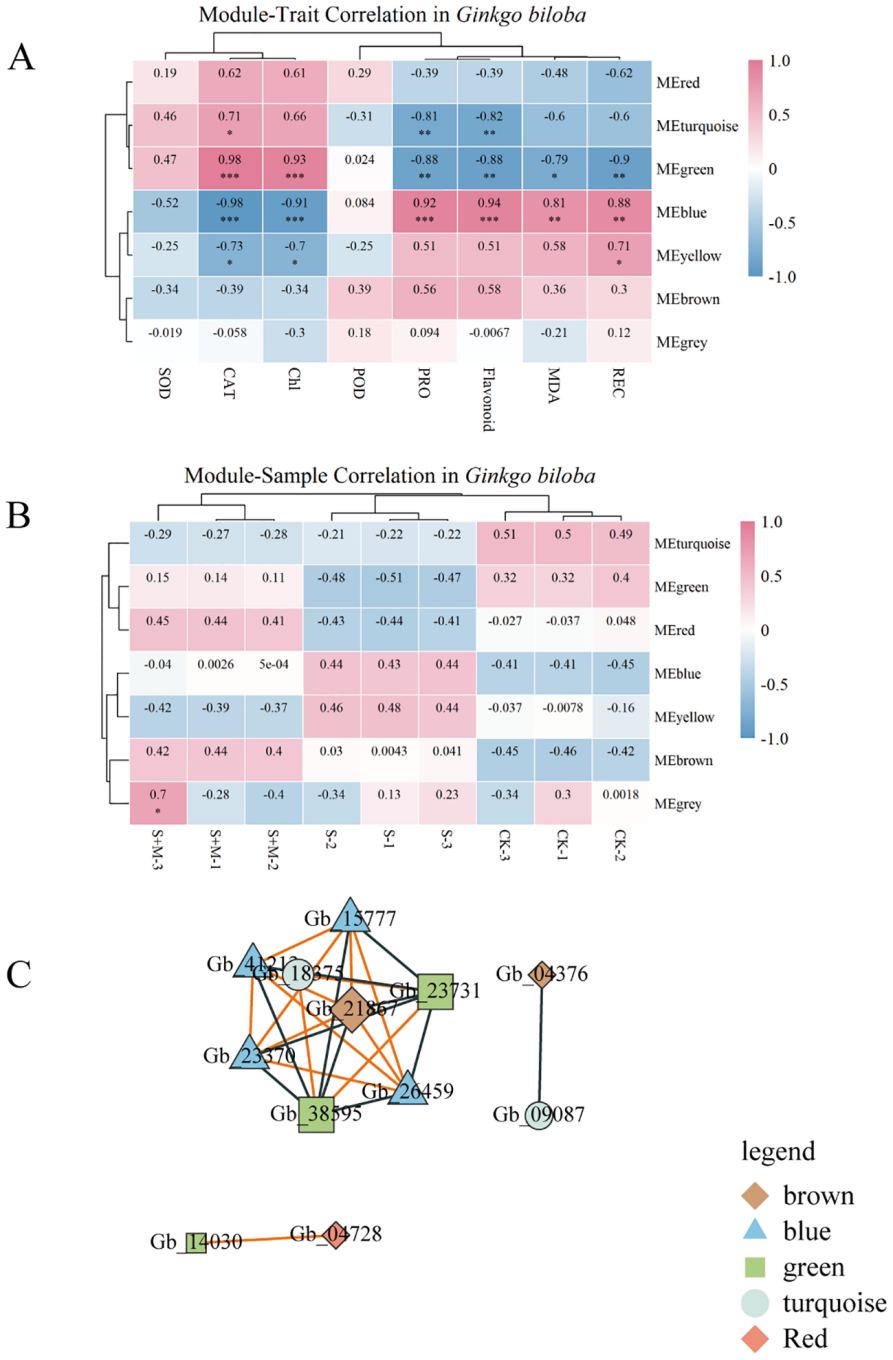
WGCNA analysis of transcriptomic data in *Ginkgo biloba* under salt stress and melatonin treatment. **(A)** Module–trait correlation heatmap showing the relationship between identified co-expression modules (rows) and physiological traits (columns). **(B)** Module–sample correlation heatmap illustrating the correlation between module eigengenes and individual samples across treatment groups. **(C)** Co-expression network of core genes involved in MAPK signaling, photosynthetic protection, and flavonoid metabolism in *Ginkgo biloba* under salt stress (*p* < 0.05, |*r*| > 0.9). Correlation colors (orange: positive; dark gray: negative) are as in **Supplementary Figure 1**. Significance levels: ***p < 0.001, **p < 0.01, and *p < 0.05. CK, S, S+M, and physiological trait abbreviations are as in **Figure 1**.

A total of six major co-expression modules were identified through WGCNA. KEGG enrichment analysis (**Supplementary Table 7**) and module–trait correlation analysis (**Figure 5A**) revealed distinct functional differentiation among the modules. MEred was predominantly enriched in pathways related to plant–pathogen interaction and plant hormone signal transduction. MEturquoise was mainly associated with metabolic pathways, biosynthesis of secondary metabolites, ribosome, and carbon metabolism, and was positively correlated with CAT activity but negatively correlated with proline and flavonoid contents. MEgreen was significantly enriched in photosynthesis, carbon metabolism, and starch and sucrose metabolism, and showed positive correlations with CAT activity and chlorophyll content. MEblue was primarily enriched in hormone signaling and MAPK signaling, and displayed positive correlations with proline, flavonoid, and relative electrical conductivity. MEyellow was enriched in biosynthesis of secondary metabolites and protein processing in the endoplasmic reticulum. MEbrown was mainly associated with biosynthesis of secondary metabolites and amino acid biosynthesis. Module–treatment correlation analysis (**Figure 5B**) indicated that MEred was positively correlated with the S+M group and negatively correlated with the S group, MEgreen was positively correlated with the CK group and negatively correlated with the S group, and both MEblue and MEyellow were positively correlated with the S group.

Based on the transcriptome data, we constructed a co-expression network using core genes involved in MAPK signaling, photosynthesis, and flavonoid metabolism (**Figure 5C**). These genes were assigned to different modules, with orange lines representing positive correlations and dark gray lines indicating negative correlations. For instance, *PP2C* (*Gb_15777*) was positively correlated with *MKK9* (*Gb_41213*), *RbohD* (*Gb_23370*), and *DFR* (*Gb_26459*), but negatively correlated with *Lhcb1* (*Gb_23731*) and *Lhcb4* (*Gb_38595*). In contrast, *ANR* (*Gb_09087*) and *F3H* (*Gb_04376*) each formed independent binary clusters with limited connections to other genes, suggesting that they may be regulated by relatively independent mechanisms.

### 3.8 qRT-PCR analysis

**Figure 6:**
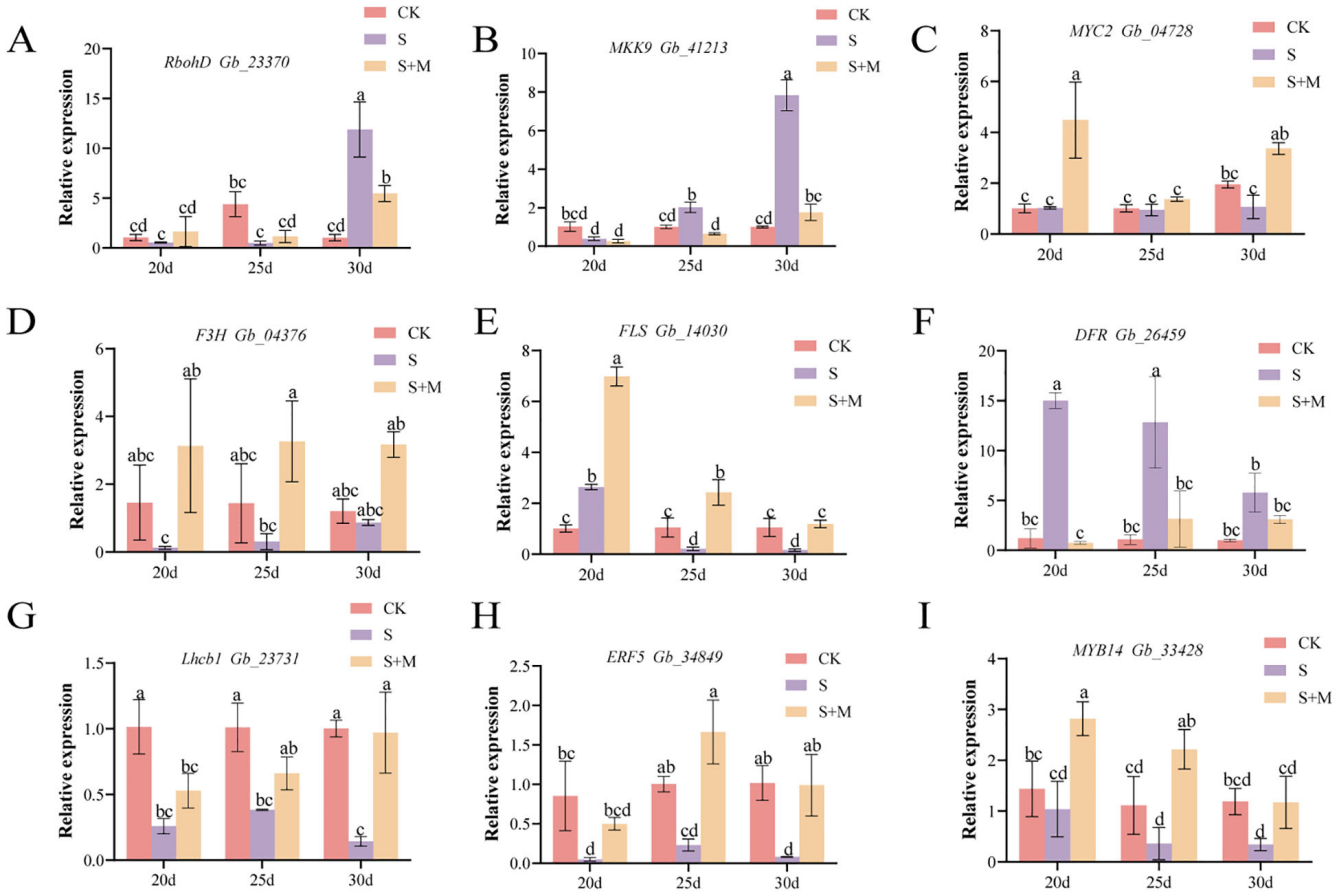
Relative expression levels of key genes under different treatments. X-axis: time points (20, 25, 30 days); Y-axis: relative expression. CK, S, and S+M are as in **Figure 1**. Different lowercase letters indicate significant differences at the same time point (Tukey’s test, *p* < 0.05). RNA-seq vs. qRT-PCR correlation at Day 30 is shown in **Supplementary Figure 7**.

To validate the reliability of the transcriptome data, nine DEGs were selected for qPCR analysis, including MAPK pathway-related genes (*RbohD*, *MKK9*, *MYC2*), flavonoid biosynthesis pathway genes (*F3H*, *FLS*, *DFR*), the PSII light-harvesting antenna protein gene *Lhcb1*, and transcription factors (*MYB14*, *ERF5*). qPCR analysis was performed on samples collected at 20, 25, and 30 days (20d, 25d, and 30d) of the 0.3% NaCl stress phase. The results showed (**Figure 6**) that at the 30 d time point corresponding to the transcriptome sequencing, the qPCR expression trends of the selected genes were largely consistent with the RNA-seq data, and the expression differences between the S+M group and the S group reached statistically significant levels (*p* < 0.05). Although the expression levels showed some fluctuations across the three time points, the overall trends were consistent with the transcriptome data, suggesting that the transcriptome data are generally reliable for further analysis. Temporal analysis revealed that *MKK9* expression in the S group continued to increase with prolonged stress duration, whereas *FLS* and *DFR* exhibited the opposite trend. Melatonin treatment differentially altered the expression levels of these genes; for instance, it significantly suppressed the stress-induced expression of *MKK9* and *DFR*, while promoting the expression of *F3H* and *MYC2*, suggesting that melatonin may regulate salt stress-responsive genes in a gene-specific manner.

## 4 Discussion

High-salt environments impair metabolic and physiological processes in plants, ultimately suppressing growth or drastically slowing development (Hualpa-Ramirez et al., 2024; Hussain et al., 2021). Elucidating the mechanisms underlying plant salt defense and developing salt-tolerant cultivars are pivotal for bolstering plant resilience under adverse environments, offering both fundamental insights and applied benefits (Zhou et al., 2024). Acting as a promising plant growth modulator, melatonin has drawn widespread interest recently owing to its functions in stimulating plant development, strengthening antioxidative systems, and maintaining ionic balance (Moustafa-Farag et al., 2020; Lerner et al., 1958). In the present study, we demonstrated that exogenous application of melatonin significantly ameliorated salt-induced foliar damage in Ginkgo biloba, as evidenced by reduced MDA content and relative electrolyte leakage, relatively stable chlorophyll levels, and enhanced activities of SOD and CAT. This trend is consistent with the report by Muhammad et al. (2025).

The MAPK signaling pathway plays a central regulatory role in plant stress responses (Lin et al., 2021; Shi et al., 2026). In this study, the expression of key MAPK pathway genes (*RbohD*, *PP2C*, and *MYC2*) was significantly altered under salt stress, and melatonin treatment partially reversed these changes. Concurrently, MDA content and relative electrical conductivity decreased in the S+M group, while proline accumulation tended toward moderate levels. These results suggest that melatonin may indirectly influence downstream oxidative stress responses and osmotic adjustment by modulating MAPK pathway gene expression. Regarding ROS regulation, RbohD generates H_2_O_2_ (the most stable ROS) and participates in the signal transduction of various plant hormones (Wei et al., 2025). Studies have shown (Kabała et al., 2022) that during the early stages of salt stress, RbohD is activated to produce H_2_O_2_ for stress signal transmission; its activity decreases during later stages, while antioxidant enzyme activity increases, jointly maintaining ROS homeostasis. In this study, melatonin treatment significantly down-regulated *RbohD* expression, which appears to be consistent with the pattern of decreased RbohD activity during later stages of salt stress to maintain ROS homeostasis. Meanwhile, the reduced MDA content and relative electrical conductivity in the S+M group suggest that melatonin may alleviate membrane lipid peroxidation damage, which could be associated with reduced ROS levels. In addition to its role in ROS production, RbohD has been implicated in the regulation of proline metabolism. Ben Rejeb et al. (2015) reported that *AtrbohD* and *AtrbohF* mutants of *Arabidopsis* exhibited significantly reduced proline accumulation under salt stress, indicating a positive regulatory role of RbohD in proline synthesis. Thus, the downregulation of *RbohD* by melatonin may concurrently contribute to both ROS alleviation and the moderation of proline accumulation observed in the S+M group. Specific PP2C members exert negative control over ABA signal transduction; functional deficiency of these proteins (e.g., *HAI*) releases the inhibition of proline biosynthesis, thus boosting proline buildup and improving osmotic adaptability (Bhaskara et al., 2012). In the present study, however, melatonin treatment down-regulated *PP2C* expression, yet proline content did not increase but rather tended toward moderate levels. This discrepancy suggests that PP2C in *Ginkgo biloba* may not act as a canonical negative regulator of proline accumulation under these conditions, or that the effect of PP2C downregulation on proline metabolism was counterbalanced by the concurrent regulation of *RbohD* and *MYC2*. On the other hand, MYC2 exhibits dual regulatory effects on proline metabolism. In plants such as Salvia miltiorrhiza and Ipomoea batatas, it can indirectly promote proline synthesis by enhancing antioxidant capacity (Deng et al., 2023; Hu et al., 2024); however, in *Arabidopsis*, it directly inhibits key genes involved in proline synthesis, thereby reducing proline accumulation (Verma et al., 2020). In this study, the up-regulation of *MYC2* was accompanied by decreased proline content, suggesting that in *Ginkgo biloba*, MYC2 might exert an inhibitory role similar to that observed in Arabidopsis. These findings provide a foundation for future functional characterization of *RbohD*, *PP2C*, and *MYC2* in proline metabolism regulation in Ginkgo biloba.

Chlorophyll content is a key indicator of plant salt tolerance and typically declines under salt stress conditions (Karimi et al., 2021). In this study, melatonin treatment slowed the decline in chlorophyll content to some extent compared with salt stress alone, suggesting a possible protective effect on the photosynthetic system. Previous work has demonstrated that PSII is more susceptible to salt stress than PSI (photosystem I) (Stefanov et al., 2024). Further analysis of genes encoding LHC proteins revealed that melatonin exerts distinct regulatory effects on the antenna systems of PSI and PSII. Specifically, the expression levels of core PSII components *Lhcb1* (*Gb_23731*) and *Lhcb4* (*Gb_38595*) were substantially restored following melatonin application, whereas core PSI members *Lhca5* (*Gb_25587*) and *Lhca1* (*Gb_18375*) exhibited more modest recovery, despite showing an upward trend. It is possible that this differential regulation helps maintain chlorophyll content and light-harvesting efficiency, which may alleviate salt-induced damage to the photosynthetic system. Furthermore, abiotic stress inhibits photosynthetic electron transport in PSI and PSII and downregulates the synthesis of photosynthetic proteins (Didaran et al., 2024). According to Takahashi and Murata (2008), Carbon fixation regulates ROS generation; when CO_2_ fixation is limited, ATP and NADPH consumption decreases, leading to an excess of NADPH, which in turn promotes electron transfer to molecular oxygen to produce H_2_O_2_. These ROS inhibit the synthesis of the D1 protein required for PSII repair, ultimately aggravating photoinhibition. In the present study, the expression of PSI- and PSII-related LHC genes was significantly downregulated under salt stress, which may represent a photoprotective response to reduce excitation pressure when carbon assimilation is limited. Following melatonin treatment, the expression of LHC family genes increased markedly, with some genes recovering to near-control levels. Concurrently, *RbohD* was significantly downregulated in the MAPK pathway, and both MDA content and relative electrical conductivity decreased, suggesting that ROS accumulation may have been alleviated. It is therefore plausible that melatonin may have contributed to the recovery of LHC gene expression, potentially alleviating transcriptional repression of photosynthetic components. However, it should be noted that transcriptional recovery of LHC genes may not necessarily reflect restored photosystem function, and whether the observed changes in *Lhcb1* and *Lhcb4* expression are due to direct regulation by melatonin or indirect effects through other photosynthetic processes remains unclear. We therefore prefer to interpret these transcriptional changes as part of the overall protective response to melatonin, without drawing strong conclusions about causal direction.

Flavonoids constitute a major class of plant secondary metabolites, encompassing subclasses such as flavonols and anthocyanins, and play a pivotal role in stress responses largely owing to their potent antioxidant capacity (Akhtar et al., 2026). Previous studies by Zu et al. (2024) have reported that melatonin promotes flavonoid accumulation. In the present study, however, flavonoid content in the S+M group was lower than that in the S group. This apparent discrepancy likely reflects that melatonin alleviates salt-induced injury through multiple pathways. It is also possible that post-transcriptional or post-translational regulation, as well as differences in metabolite turnover or degradation rates, may contribute to the inconsistency between gene expression levels and actual flavonoid accumulation. Specifically, melatonin treatment helped preserve chlorophyll content, appeared to protect the photosynthetic apparatus, and was associated with reduced MDA levels and relative electrolyte leakage, suggesting mitigated membrane lipid peroxidation. Under these ameliorated stress conditions, the overall cellular stress burden appeared to be reduced, which may have contributed to the relatively lower flavonoid accumulation observed in the S+M group.

Co-expression network analysis linked MAPK signaling, photosynthetic protection, and flavonoid metabolism at the module level. MEblue (MAPK-enriched) was positively correlated with PRO, flavonoids, REC, and salt stress, whereas MEgreen (photosynthesis-enriched) showed positive correlations with CAT and chlorophyll but negative correlation with salt stress. These results appeared to be generally consistent with the physiological phenotypes: MEblue appeared to be activated under salt stress, MEgreen was suppressed, and melatonin treatment seemed to restore MEgreen expression, which was associated with the reduced MDA and REC and increased chlorophyll observed in the S+M group. The core network further suggested that MAPK-related genes (*PP2C*, *MKK9*, *RbohD*) clustered with the flavonoid gene DFR, but tended to show opposite trends to *Lhcb1* and *Lhcb4*, suggesting that stress signaling and photosynthetic suppression may occur together under salt stress, and that melatonin might partially reverse this pattern. Overall, melatonin appears to act through coordinated regulation of multiple modules rather than a single pathway, potentially helping G. *biloba* adapt to salt stress.

## 5 Conclusions

**Figure 7:**
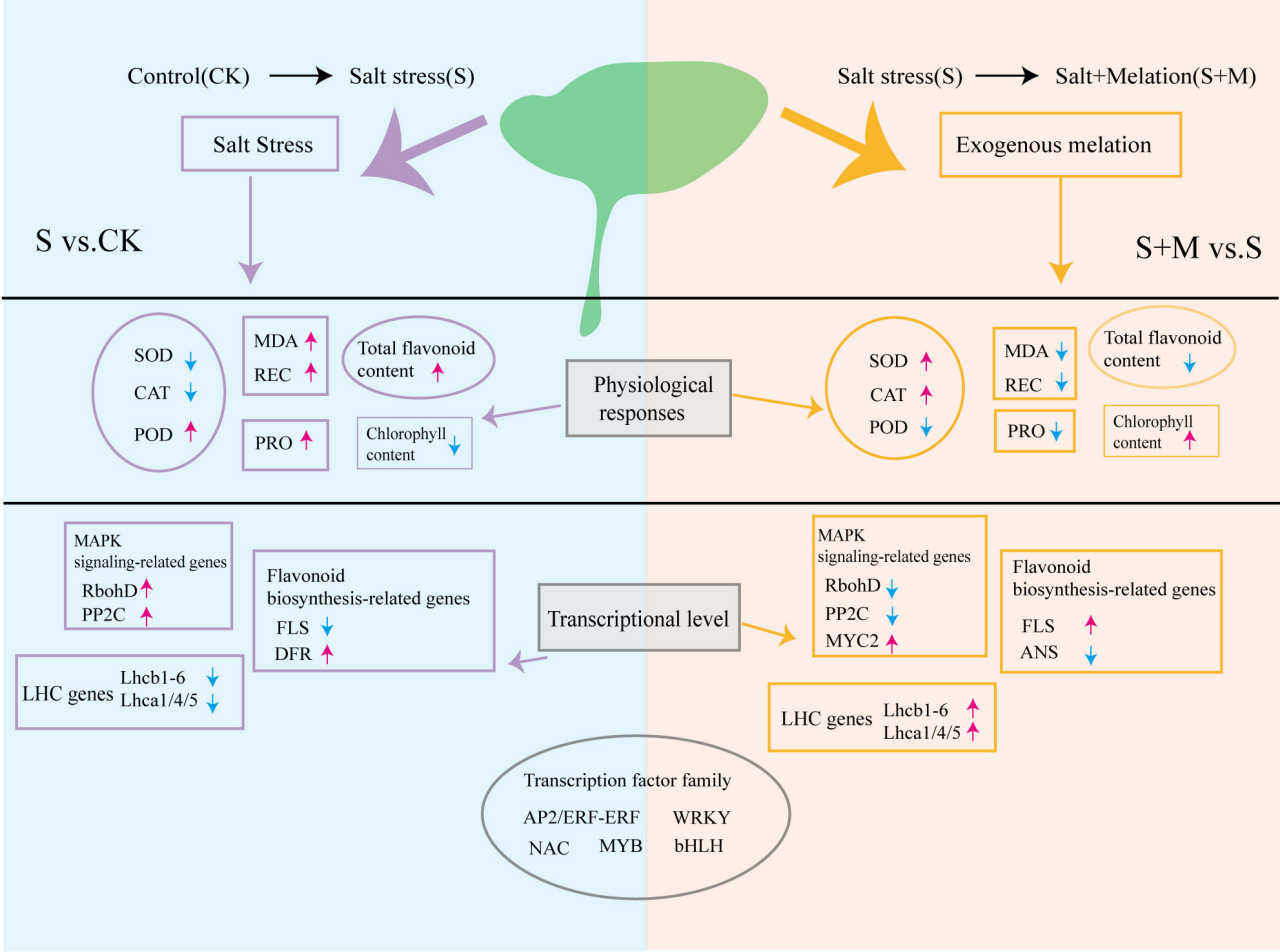
Hierarchical summary of physiological and transcriptomic responses. Left panel (blue): S vs. CK (purple: salt stress); right panel (orange): S+M vs. S (yellow: melatonin). Pink arrows: upregulation/increase; blue arrows: downregulation/decrease. Upper layer: physiological responses (SOD, CAT, POD, MDA, REC, PRO, chlorophyll, flavonoids). Lower layer: transcriptional changes (MAPK: *RbohD*, *PP2C*; Flavonoid: *FLS*, *DFR*, *ANS*; LHC: *Lhcb1-6*, *Lhca1/4/5*). No direct causal relationships are implied between layers. Transcription factor families (AP2/ERF, WRKY, NAC, MYB, bHLH) were differentially expressed. CK, S, S+M, and physiological abbreviations are as in **Figure 1**. Abbreviations for RbohD and PP2C are as in **Supplementary Figure 4**; Lhcb1–6 and Lhca1/4/5 as in **Supplementary Figure 5**; FLS, DFR, and ANS as in **Figure 4A**.

Under salt stress, *Ginkgo biloba* leaves showed reduced chlorophyll content and downregulation of LHC family gene expression, suggesting a potential impairment of photosynthetic capacity. This was accompanied by increased MDA levels and relative electrical conductivity, suggesting possible aggravated membrane lipid peroxidation. Transcriptomic analysis revealed altered expression of genes involved in the MAPK signaling pathway and flavonoid biosynthesis pathway, as well as substantial proline accumulation, which may contribute to reduced salt tolerance. Exogenous melatonin partially alleviated these effects. Melatonin appeared to modulate key MAPK pathway genes (*RbohD*, *PP2C*, and *MYC2*), which may help restore ROS balance and regulate moderate proline accumulation, accompanied by reduced membrane damage. In addition, melatonin appeared to help restore the expression of PSII-related LHC genes and helped stabilize chlorophyll content, while reducing flavonoid accumulation. Collectively, melatonin appears to alleviate salt-induced damage in *Ginkgo biloba* by coordinately regulating physiological and molecular responses, potentially contributing to enhanced salt tolerance. The schematic illustration is presented in **Figure 7**.

Functional validation of the candidate genes identified in this study will be an important direction for future research, which will help to further elucidate the molecular mechanisms underlying melatonin-mediated salt tolerance in *Ginkgo biloba*.

## Data Availability

The raw RNA-seq data supporting the conclusions of this article have been deposited in the NCBI Sequence Read Archive (SRA) under BioProject accession number PRJNA1474691. https://www.ncbi.nlm.nih.gov/bioproject/PRJNA1474691.
